# Manipulating Pixels in Computer Graphics by Converting Raster Elements to Vector Shapes as a Function of Hue

**DOI:** 10.3390/jimaging9060106

**Published:** 2023-05-23

**Authors:** Tajana Koren Ivančević, Nikolina Stanić Loknar, Maja Rudolf, Diana Bratić

**Affiliations:** Faculty of Graphic Arts, University of Zagreb, 10000 Zagreb, Croatia

**Keywords:** pixel shape, hue, color systems, computer graphics, security graphics

## Abstract

This paper proposes a method for changing pixel shape by converting a CMYK raster image (pixel) to an HSB vector image, replacing the square cells of the CMYK pixels with different vector shapes. The replacement of a pixel by the selected vector shape is done depending on the detected color values for each pixel. The CMYK values are first converted to the corresponding RGB values and then to the HSB system, and the vector shape is selected based on the obtained hue values. The vector shape is drawn in the defined space, according to the row and column matrix of the pixels of the original CMYK image. Twenty-one vector shapes are introduced to replace the pixels depending on the hue. The pixels of each hue are replaced by a different shape. The application of this conversion has its greatest value in the creation of security graphics for printed documents and the individualization of digital artwork by creating structured patterns based on the hue.

## 1. Introduction

There has been a desire to modify and refine digital pictures since the beginning of computer graphics and even today. This work was inspired in part by a series of articles published in the 1990s, which dealt with the artistic rasterization (screening) of images [1,2]. In these papers, artistic screening was described as a new image reproduction technique in which freely designed screening elements were used to create halftones. These were black and white images. In artistic screening, the shape of the screen dots can be customized according to the designer’s will. Various screen shapes created in Adobe Illustrator were used. They are used in the creation of graphic designs of high artistic quality and in the field of security graphics [3]. In addition, the development of computer graphics allows for the research and creation of mathematical tools for computer-generated ornamental patterns to create images [4]. To improve image quality without increasing the resolution of the square pixels, variable shaped pixels are used [5,6]. The Mathematica program can be used to test the output shapes of future screen elements. By translating mathematical functions into the Postscript programming language, a database of screen elements of various shapes was developed and created [7,8]. Experiments with variable shapes of screen elements, so-called mutants, have also been performed [9]. Multicolor dithering is applied to generate color images whose screen dots consist of artistic shapes (letters, symbols, ornaments, etc.) [10]. An algorithm is also presented for extracting a resolution-independent vector representation from pixel art images, which can be used to arbitrarily enlarge the results without image degradation [11].

Images can be manipulated by applying various filters that enhance certain properties of the image. In particular, the application of the Gaussian filter is frequently cited in the literature as a basis for further research [12,13,14,15]. The Gaussian filter is an important component of many algorithms in image processing. The bilateral filter was originally introduced for the task of image denoising as an edge-preserving filter [16,17,18,19]. There are works in which joint bilateral filtering improves the quality of photographs by combining an image taken with daylight and one taken with flash [20,21]. Trilateral filters for high contrast images and meshes built from two modified forms of Tomasi and Manduchi’s bilateral filter for edge-preserving smoothing and visual detail removal for n-dimensional signals in computer graphics, image processing, and computer vision applications have also been presented [22].

One of the most important and commonly used manipulations of images is pseudocoloring, which has applications in various fields. There are several techniques for coloring satellite images SAR. Deep learning has become the main method of SAR colorization, with the most advanced pix2pix method [23]. One of the methods is based on transferring black and white images to the RGB system through the CIE Lab system [24]. Various techniques are also used to enhance the colors in the images. For example, there is a method to improve saturation in the context of hue-preserving color image enhancement in an RGB color space. The proposed method handles colors in an RGB color space that has the shape of a cube, and improves the contrast of a given image by histogram manipulation [25]. There are also methods to improve contrast by reversible data hiding [26].

In the field of security graphics, various methods are used to protect the originality of the image. There are various techniques to secure digital images such as encryption, steganography, and watermarking [27,28,29]. Using fractal geometry, Hilbert curve-based image protection software has been developed in Postscript programming language [30].

However, to our knowledge, there are no scientific studies in which the appearance (shape) of the pixels themselves has been changed. Our research provides a suggestion on a method to change the shape and appearance of pixels by inserting the various vector shapes into an empty pixel cell. For this purpose, different vector shapes have been designed to represent pixels in different ways for each hue in the HSB color space. The aim of this research is to show that it is possible to change the appearance of each pixel and thereby affect the appearance of the entire image on the basis of the hue value. In the results of the research, we were able to present a multi-color graphic in which a different shape is automatically displayed for each hue instead of the original pixels.

## 2. Materials and Methods

According to CIE, colors are represented geometrically in space, usually in 3 dimensions. Color spaces are divided into device-dependent and device-independent spaces, according to the 1996 IFRA Special Report. Device specific color spaces represent colors relative to device properties. The most commonly used device-dependent color spaces are RGB, CMY, CMYK, and YCC color spaces. Device-independent color spaces are based on consistency with a standard observer’s perception, as well as perceptual uniformity. The most common ones are: HSB, CIE XYZ, CIE xyY, CIE Lab, CIELAB, and CIE Luv. In this document, the color systems CMYK, RGB, and HSB are used [31].

CMYK is a subtractive color system whose primary colors are cyan, magenta, and yellow, with black added. In this system, also known as “printer inks”, colors are viewed from the perspective of the reflection of white light on a surface. The color obtained is the result of subtraction from the white color. Theoretically, if all the colors in this system are mixed together, the result is the color black. The values of the CMYK colors range from 0 to 100% [32].

The additive or RGB color model consists of three primary colors: red, green, and blue. The RGB model is used by RGB monitors and LCD screens. When all three components are added together, white is (theoretically) obtained. The color values in the RGB system range from 0 to 255 [33].

The HSB color model is a model of secondary and complementary colors. In it, the combination of primary colors from the two previously mentioned systems leads to all other colors or tones. The color wheel is used to represent hues and their ‘positions’. The hue is given in degrees, and the values for the primary colors of the additive color model can be easily determined by dividing 360 degrees into thirds (R = 0 or 360, G = 120, B = 240). By combining the primary colors of the additive color model, the primary colors of the subtractive color model are also determined (R + G = Y = 60, G + B = C = 180, B + R = M = 300 degrees). In this way, all other color tones can be defined. Moreover, each primary color of the additive system is complementary to one color of the subtractive system (R~C, G~M, B~Y). Saturation is a property which defines full hues or shades of gray, while brightness controls the lightness or darkening of the color [34,35,36,37].

**Figure 1:**
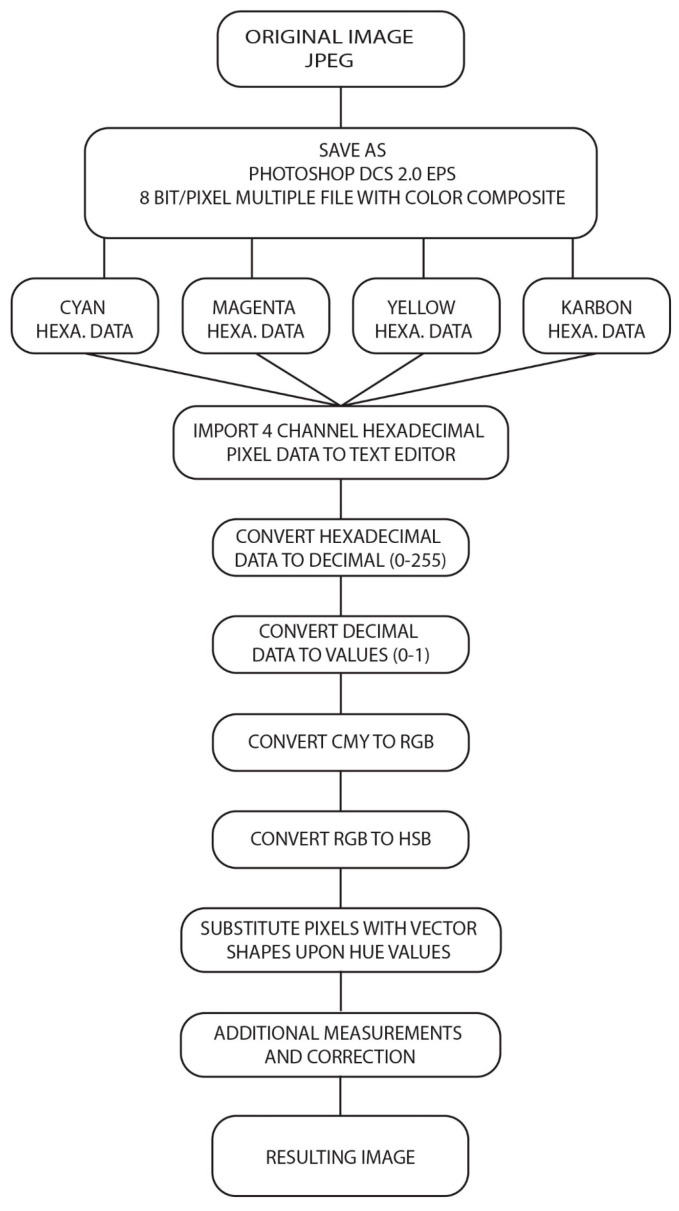
Translation of the original image in jpeg to resulting image with shapes defined by color hue.

In this research, the Postscript programming language is used for image analysis and pattern creation [38,39,40,41]. Instead of classical halftone elements, individual shapes are created whose position is defined by the location of the pixel cell in the coordinate space of the image. The shapes are first sketched and then programmed. A square cell is created in which new vector shapes are programmed. The shapes are programmed to use data about the dimensions of a particular square shape, which is the pixel size, so they can easily be sized in relation to the dimensions of the original pixel. To apply new vector shapes, graphics with division into CMYK system channels are used. Each image channel is defined by a hexadecimal matrix, and the color component of each pixel is described by two values from 00 to FF. Converted to the decimal system, it is possible to represent 256 hues for each CMYK channel. The hue of each pixel is determined by representing all four components of the CMYK channels. The original hexadecimal code of the image is translated into the decimal system and normalized (where 0 represents the absence of color and 1 represents the maximum tone intensity). Figure 1 shows the research plan of image transformation from the original image in jpeg format to the image with applied vector shapes based on the hue value.

The algorithm reads the CMYK values of each pixel and converts them to values between 0 and 1. The rows and columns of the matrix are defined programmatically, based on the image width and height. To correctly represent the pixels from all four CMYK channels, a pixel width offset is defined on the x-axis for the columns and a pixel height offset is defined on the y-axis for the rows. It is important to emphasize that in Postscript, it is possible within one program (one printable page) to use all three-color systems—CMYK, RGB, and HSB—simultaneously. This possibility will prove to be very necessary when continuing the experimental part of the work.

Algorithm for creating a matrix of cells based on the image pixel position:

- Define the width (w) and height (h) of the cell at the position of the original pixel;
- Define the total number of columns (S) and rows (R) of the matrix;
- Construct a matrix in which the cell positions x and y are defined as x = w × s; y = h × r, where ‘s’ and ‘r’ are the values of the current column and row.

**Figure 2:**
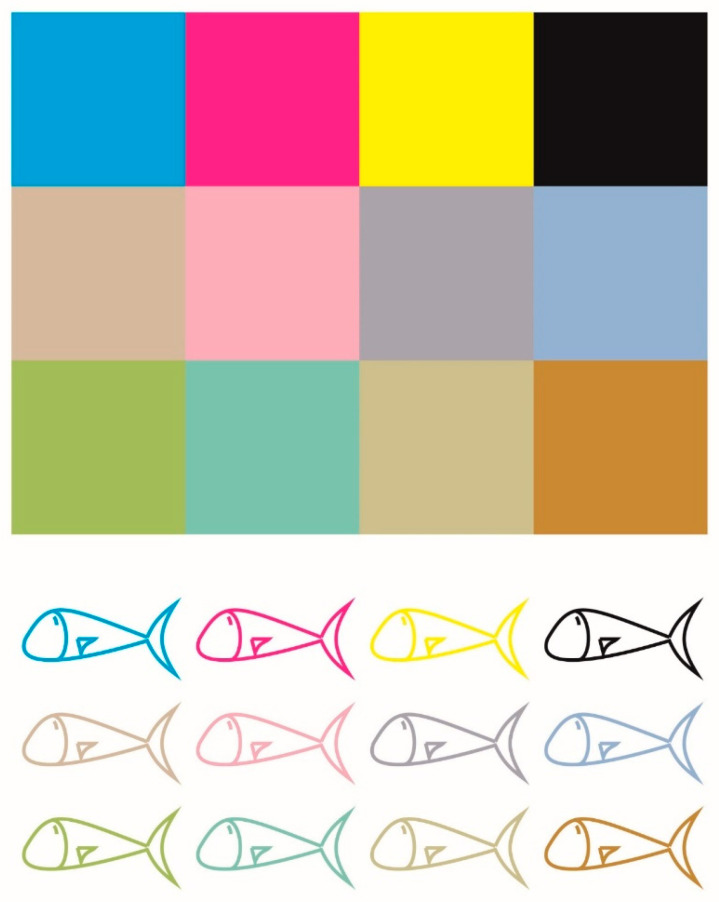
Shows 12 pixels of different hues and their replacement with a new fish shape.

When a cell is created with its own dimensions and position, an arbitrary shape is created that defines a new raster element with the dimensions of the cell itself. This raster element contains information about the coloring that was previously associated with the pixel. The plan for conducting the experiment is shown in Figure 2.

For the first phase of the research, an 8-bit raster image is used, consisting of 3 rows and 4 columns. In the first example, four solids of the CMYK system were used in the top row, while different hues were mixed in the other two rows. The color data are expressed in hexadecimal values, and their image is displayed with these values, i.e., an image of 12 pixels with different hues. The same image was used to manipulate the pixels that now represent the vector shape of the “fish”. The vector shape is placed in the previously defined square shape with pixel width and height. The data of each color channel are converted from hexadecimal values to values between 0 and 1, adapted to the Postscript programming language. The newly obtained data for each pixel are then plotted in the shape of a fish.

**Figure 3:**
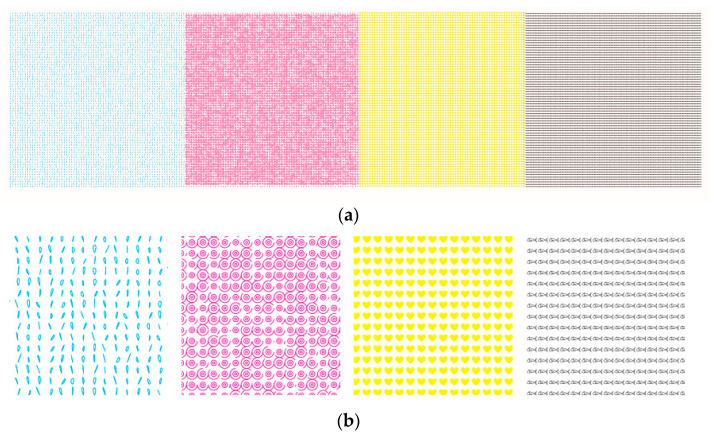
(**a**) Shows four colors where the original pixels are replaced by four new shapes, and (**b**) shows enlarged details of the pixel layout.

In the next phase of the research, a different shape is used for each of the CMYK channels. The experiment consists of four solid squares of C, M, Y, and K. The image is saved with the separation of the channels. A new alternative pixel shape is defined for each channel (Figure 3a,b).

The pixels in the cyan channel are replaced by a Bezier curve to which a pseudorandom number has been added as the x value in the first control point and the x and y values in the second control point. In this way, the curves are drawn differently for each pixel, randomly but predefined by the seed of the pseudorandom numbers.

In the magenta channel, the pixels are replaced by three circles, with the initial angle at the largest of the three circles also determined by pseudorandom numbers.

The pixels in the yellow channel are replaced by heart shapes.

In the last K channel, the pixels are replaced by a fish shape. The conditions are set to split the image so that a different replacement shape for the pixels is chosen for each channel.

**Figure 4:**
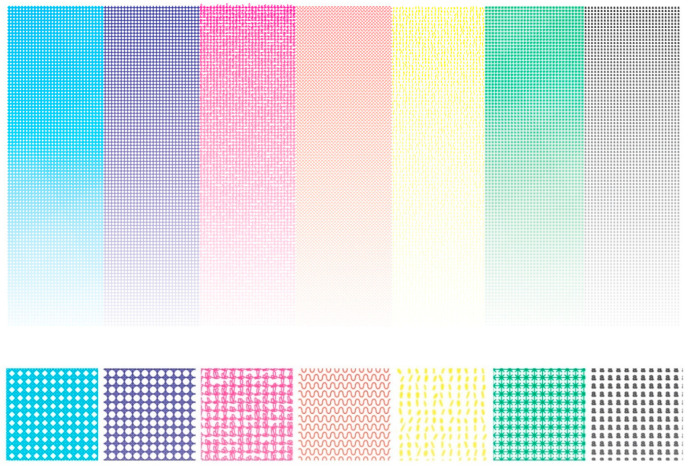
Four basic colors of the CMYK system and three basic colors of the RGB system in which the new shapes replace the original pixel shape.

In the continuation, the investigation has been extended to the three basic colors of the RGB system (Figure 4). Seven new shapes were used: random curves (lines), the letter ‘a’, a star, a wave, a diamond, and rotating squares.

Seven new shapes were used: random curves (lines), the letter ‘a’, a star, a wave, a diamond, and rotating squares (Figure 4).

**Figure 5:**
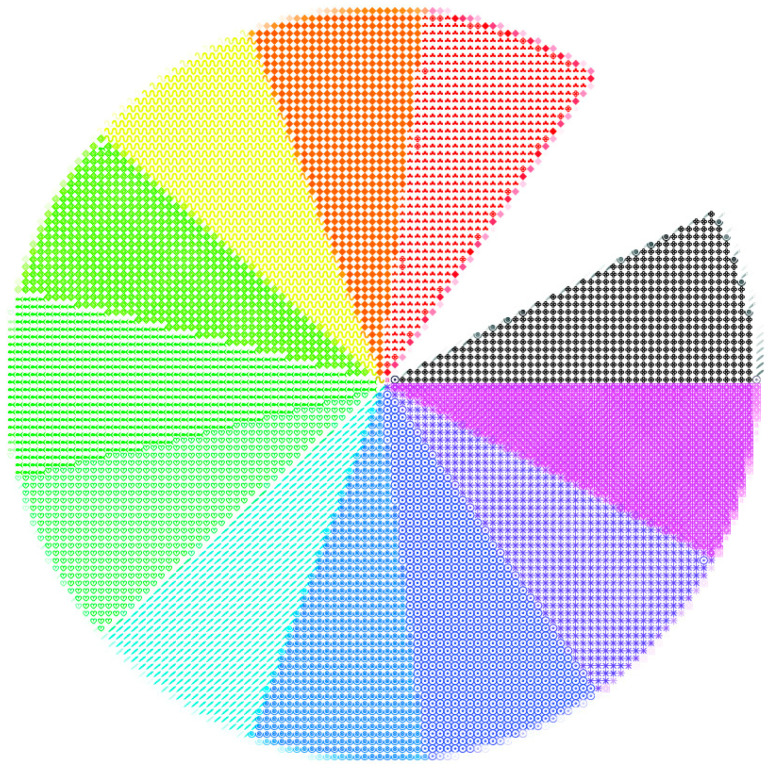
Shows the pixels replaced by vector shapes for 11 hues by the HSB system and 1 for black.

In the next phase of research, we wanted to extend the sampling of new replacement shapes for the classic pixels to more hues, not just the basic ones from the CMYK and RGB systems, for which it is easy to set the conditions. We encountered an obstacle in that it was not possible for us to achieve an appropriate response by combining these two-color systems and setting conditions for selecting another element for a particular hue. Setting the condition would result in at least one hue popping out and displaying the wrong vector element or no vector element at all. Therefore, the conversion was made from the CMYK system to the RGB system to the HSB system. Figure 5 shows the result of displaying different hues after conversion.

The first step is to read the cyan, magenta, yellow, and black values from the hexadecimal data contained in the image.

To perform the conversion from CMYK to RGB, the CMYK values read from the image are converted to CMY (K = 0) using the “gray component replacement” (GCR) method with the minimization of the black component.

The formulas in Equation (1) show the conversion from CMYK to CMY (K = 0):

C1 = C + K M1 = M + K Y1 = Y + K K1 = K − K = 0(1)

In the next step, the values of the newly obtained C, M, and Y are converted to the RGB system using the complementarity method shown in Equation (2):

R = 1 − C1 G = 1 − M1 B = 1 − Y1(2)

The last step is the conversion from RGB to HSB. First, the largest and smallest values of the RGB components must be determined (e.g., for R = 25, G = 70, B= 15, the smallest value would be the B component and the largest would be the G component). The highest value ‘max’ is appended to the brightness component of the HSB.

Hue and saturation are defined by the difference between the maximal and minimal RGB value. If the difference is zero, then the saturation is also zero, otherwise it is calculated as follows in the formula:

S = (max − min)/max(3)

For hue, the following conditions apply: If the difference Δ_max–min_ between the max and min is zero, then the hue is zero. If this is not the case, then the highest value of the three RGB components is searched for (max) and calculated according to the following expressions in Equation (4):

For R = max, H = (G − B/Δ_max–min_) × 60/360 For G = max, H = 2 + (B − R/Δ_max–min_) × 60/360 For B = max, H = 4 + (R − G/Δ_max–min_) × 60/360 (4)

if H turns out to be a negative number in a calculation, 1 is added to it.

**Figure 6:**
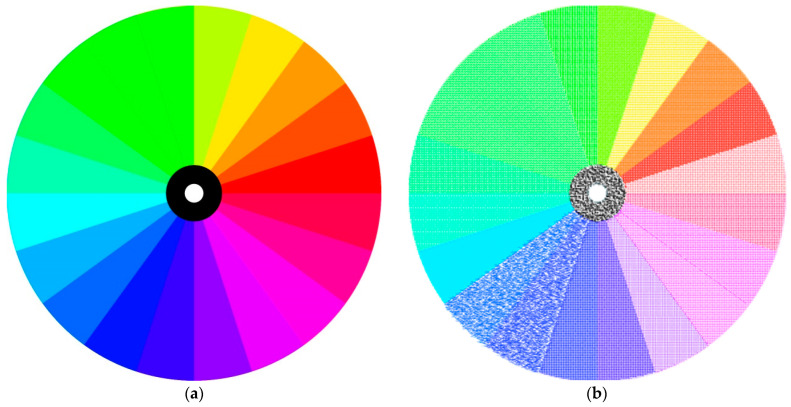
(**a**) Showing the color wheel divided into 20 hues and (**b**) the result obtained by replacing the pixels with vector shapes.

**Figure 7:**
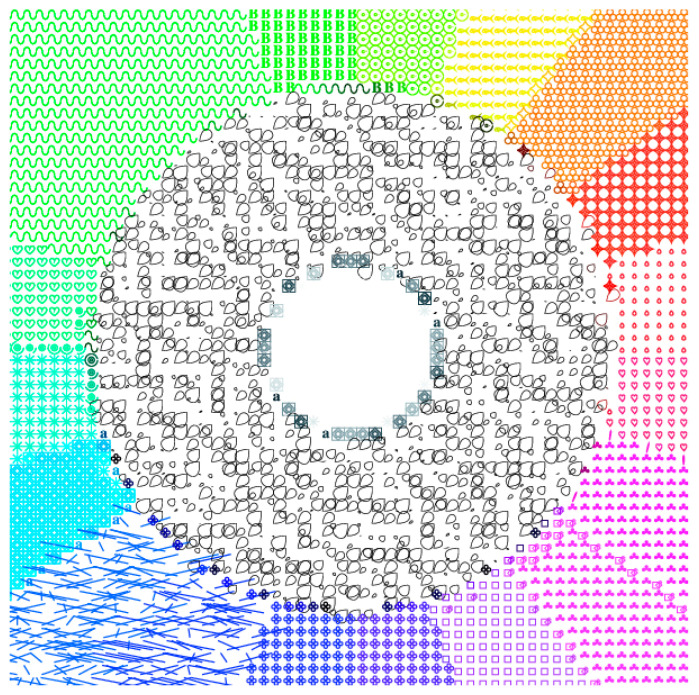
Shows enlarged details of Figure 6b. Some hues responded correctly to the program’s requirements, others did not. Some shapes appear in more than one hue interval (green waves, blue lines).

This experiment led to the final phase, where a problem was also encountered. The HSB color wheel was used, which is divided into 20 hues. The pixels of each hue were to be replaced by a vector shape. However, while some hues responded correctly to the program’s requirements, others did not. The result is shown in Figure 6b and Figure 7 (enlarged detail of Figure 6b). Figure 6a presents the original image that was used totest our method. The Postscript code of this example can be found in the supplementary material under the name Code S1.

## 3. Results

**Chart 1:**
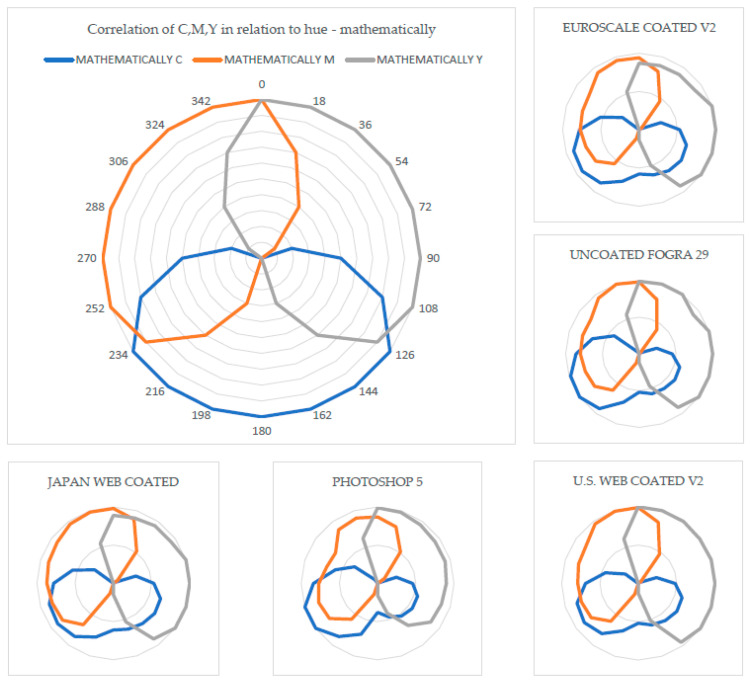
Shows the values from Table 1 for the first four columns, i.e., the correlation of C, M, Y as a function of hue, the mathematically determined values (**large image**), and the values obtained by measurement for different color settings (**5 small images**).

Since the shapes were not distributed according to the hues and conditions set, additional measurements and corrections were made. It was found that the color settings affect the way of conversion from the CMYK system to the HSB system, and also the distribution of the shapes within a certain interval. The colors are grouped around some tones, i.e., they are too similar to each other, and this is exactly the reason why the program uses the same shape for several neighboring tones, despite the precisely set conditions (Figure 7). The measurements gave different results. The hues group around certain hue angles. This is particularly pronounced for green, green-blue, blue, and blue-violet hues, hue (72, 90), hue (90, 108, 126, 144), hue (234, 252), hue (252, 270), and the general absence of cyan (hue 180). Although the hues constantly increase by a constant angle of 18 degrees, in reality, the C, M, and Y values do not exactly follow this change. For example, at angles 90, 108, 126, and 144, the equivalent of the C, M, and Y values are almost the same (53, 0, 100; 65, 0, 100; 68, 0, 100; 66, 0, 91). After extensive analysis and measurements, we concluded that the hue spacing must be increased for certain hues and decreased for others to achieve an appropriate distribution of hues across all twenty parts and black. To produce the desired hue separations, five different color settings were used to analyze the hue behavior of C, M, and Y. The hue of each of the five color settings was then used to produce the desired hue separations. These color settings provide different separation methods and are designed to use different inks under different printing conditions and on different papers. Euroscale Coated V2, for example, uses specifications designed to produce high-quality separations with Euroscale inks at 300% total ink coverage, positive plate, and bright white coated paper. Fogra was developed by the German Graphic Technology Research Organization, Japan was developed by the Japan Magazine Publisher Association, and the U.S. was developed to produce quality separations using U.S. inks. Using these color settings and the differences in the C, M, and Y values in correlation to hue, as shown in Table 1, will result in the correct color setting and the correct distribution of shapes as a function of hue. The first column of Table 1 contains the data used for the example in Figure 7. The other columns contain measurement data as a function of various color settings. Chart 1 shows the poor distribution of C, M, and Y in certain parts of the spectrum. Table 1 shows the C, M, and Y values correlated with the hue angle. Viewed from left to right, the mathematically calculated values are shown first, followed by the measured values using five different color settings.

Chart 1 shows the values from Table 1 for the first four columns, i.e., the correlation of C, M, and Y as a function of hue. The mathematically determined values (large image) show the equal distribution of the values. The values obtained by the measurement for different color settings (five small images) show how the measured values deviate from the mathematical model. The greatest changes are visible in the cyan (C) and magenta (M) values, while the yellow (Y) values remain close to the mathematical model.

By correcting the interval, the correct distribution of shapes was achieved by the hue spectrum, which was divided into 20 parts, and the added black component, which was the goal of this research. 

**Figure 8:**
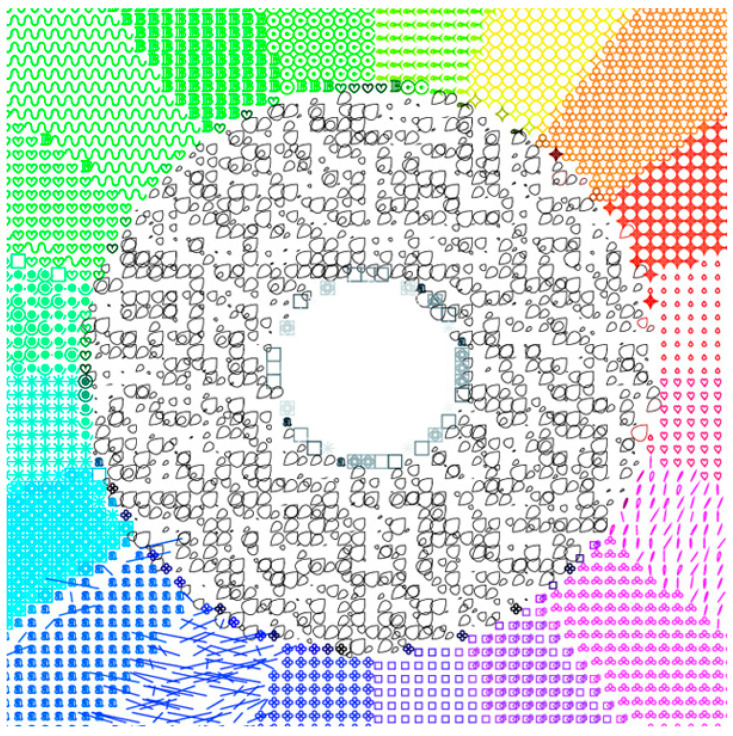
Showing a color wheel with 21 different shapes (20 + black) replacing the original pixels with corrected intervals of hue values.

The final result is shown in Figure 8. All 21 shapes were used, each in its own segment or interval. It can also be seen that individual elements appear in other segments, as already shown for the most challenging blues and greens.

**Chart 2:**
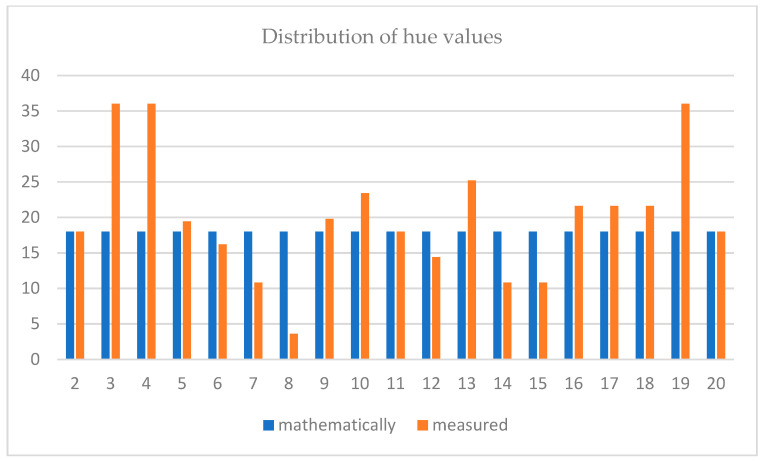
The comparison of the distribution of hue values obtained mathematically by dividing the circle into 20 equal parts (delta) and hue intervals for correctly displayed shape distributions. Table 2 shows the values obtained mathematically at the beginning, with intervals of 18 degrees and the values for the correct representation of the shape by the individual segments.

Figure 7 shows variations in individual hue intervals where the program did not use different shapes for different intervals, but used the same shape to represent several adjacent intervals. After correcting the tones, the result shown in Figure 8 was obtained so that all 20 tones and the black color are displayed. The Postscript code of Figure 8 can be found in the supplementary material under the name Code S2. Chart 2 shows the deviations of the values of each hue interval from the values obtained by calculation. The differences in degrees between the initial and final angles within which a shape or hue value is displayed for both cases are shown. The correct distribution of the difference values of 18 degrees is shown in blue, and the visibly fluctuating difference is shown in red.

**Chart 3:**
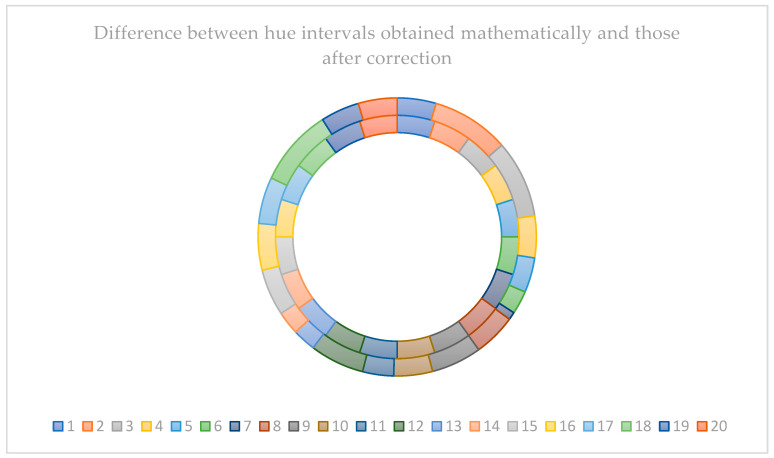
The difference between the mathematically determined hue intervals (inner circle data—intervals of 18 degrees) and those after correction showing the result of our method (outer circle data—different hue intervals). Chart 4 shows the difference between the initial and final values of the hue interval determined by the program and those measured in the case of a regular shape distribution on the color wheel. Since the differences between the initial and final values of the hue interval in the program solution are always 18, straight lines (blue and orange) are obtained, while oscillations (gray and yellow) can be seen in the corrected values.

Chart 3 not only shows the difference between the calculated intervals and those after correction, but also the possibility to visualize parts of the spectrum. The difference with the color wheel example (Figure 8) is that the distribution of colors and shapes in the example starts at 90° (3 of the clock) and moves counterclockwise, while in Graph 3, it starts at 0° (12 of the clock) and moves clockwise.

**Figure 9:**
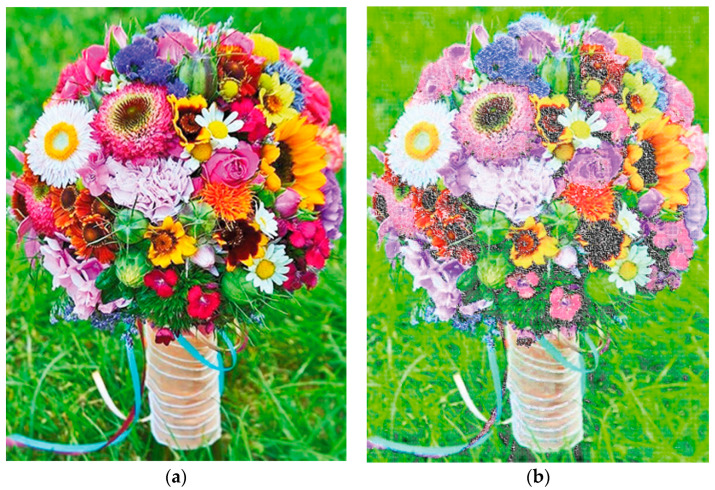
(**a**) Original image of colorful bouquet of flowers (Source: https://i.pinimg.com/originals/92/a5/3f/92a53f294d9309d526e2469765028a75.jpg, visited on 29 April 2023) and (**b**) the resulting image after the pixels were replaced by different shapes based on the detected hue values.

**Figure 10:**
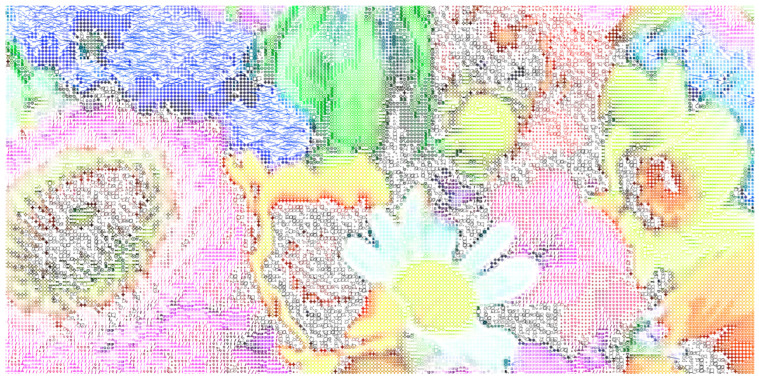
The enlarged details of Figure 9.

Once the proper distribution of shapes is determined according to a particular hue, the search is extended to color images. The image of a colorful bouquet of flowers is used in the next example, as shown in Figure 9a. Figure 9b shows the result according to the distribution of shapes depending on the determined values of the hue for each pixel. Figure 10 shows the enlarged details of Figure 9.

**Figure 11:**
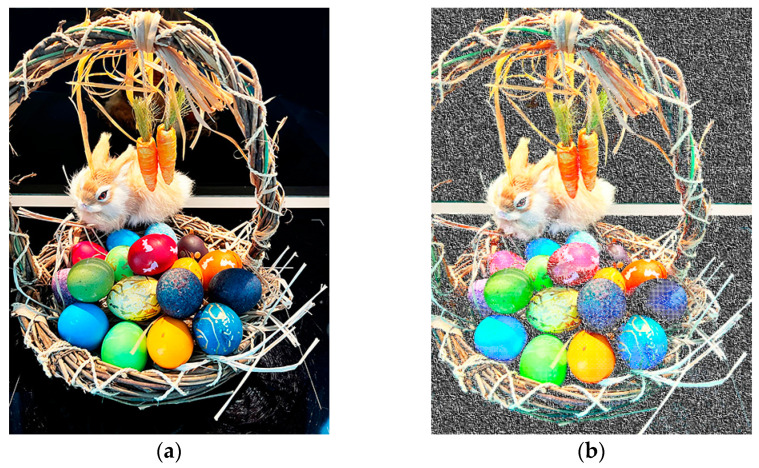
The image of Easter eggs. (**a**) Shows the original image and (**b**) shows the result after applying our method.

**Figure 12:**
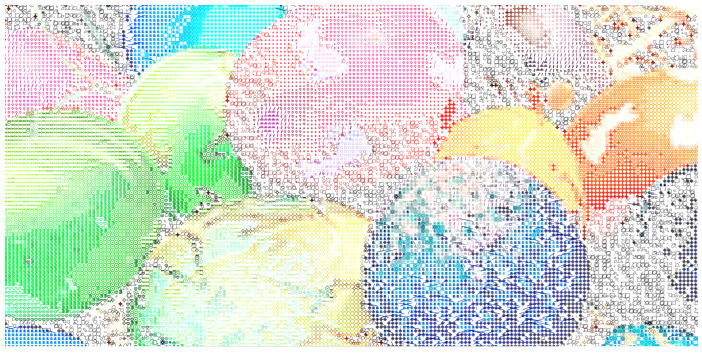
Shows the enlarged details of Figure 11.

The next example used in our research is the image of Easter eggs (Figure 11). Figure 11a shows the original image, while Figure 11b shows the result after applying our method. Figure 12 shows the enlarged detail of the Figure 11 b).

**Figure 13:**
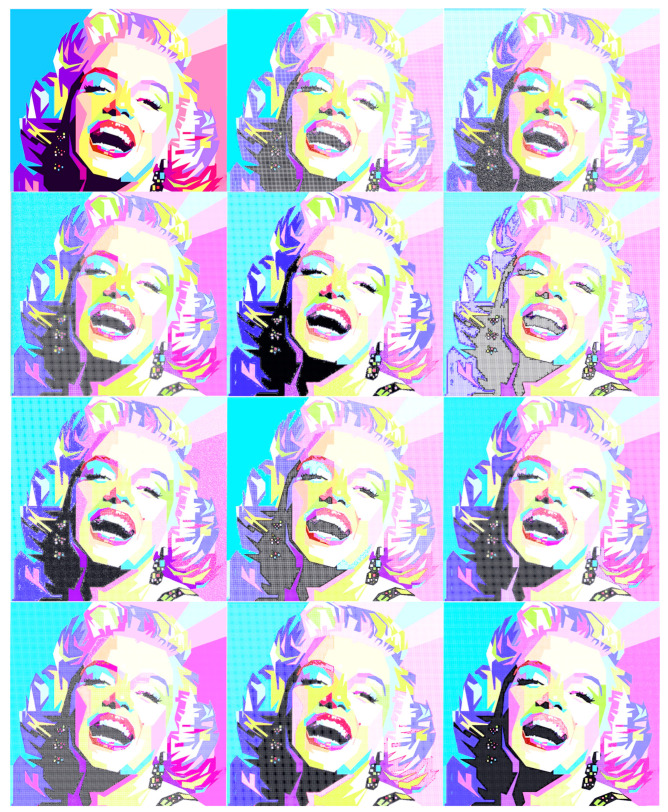
Proposed method implemented on pop art image of Marilyn Monroe (Source: https://www.pxfuel.com/en/desktop-wallpaper-eejre, visited on 2 May 2023), creating numerous different solutions based on the change of shapes for the same hue.

In addition to real nature images, we also tested our method on pop art images of Marilyn Monroe. Figure 13 shows the artwork obtained by changing the shapes for the detected hue. In this way, numerous completely different results can be obtained. In this example, there is no right or wrong shape distribution. It is a matter of taste as to which looks better than the other.

**Figure 14:**
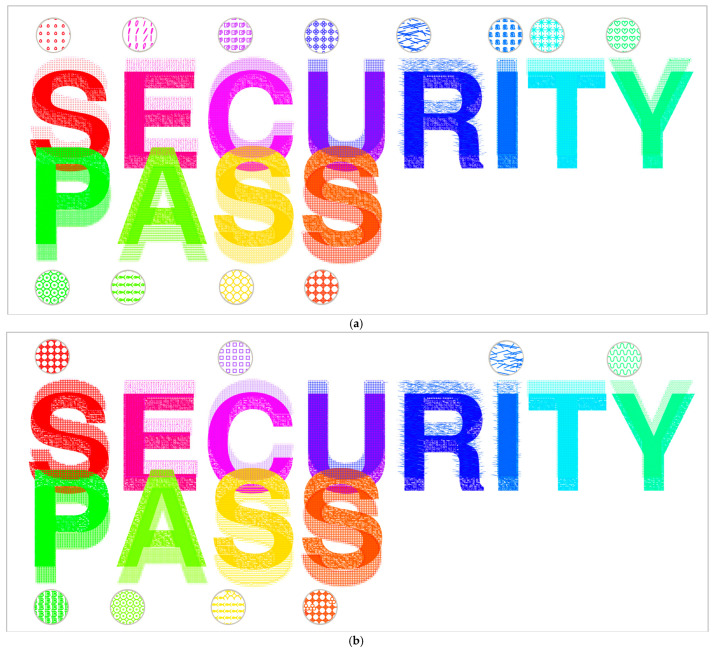
(**a**) Right distribution of the shapes obtained with our method. (**b**) The distribution of the shapes obtained using Uncoated Fogra 29 color setting—deviations are visible in the letters S, C, I, Y, P, A, S, S.

The next example shows the difference in selecting shapes by detected hue on images created with different color settings (Figure 14). Figure 14a shows the distributions of the shapes depending on the detected hue using our method, while Figure 14b shows the deviations in shape distribution using color setting Uncoated Fogra 29. Additional figures can be found in the supplementary material under Figures S1–S4. The figures show the deviations of the shape distribution depending on the detected hue for different color settings as follows: Figure S1: Euroscale Coated V2; Figure S2: Japan web coated; Figure S3: Photoshop 5; and Figure S4: U.S. web coated V2. This example also shows that even using the original graphics for protective printing can lead to an incorrect result. Using an incorrect color setting can result in different shapes being displayed that represent pixels for a detected hue.

**Figure 15:**
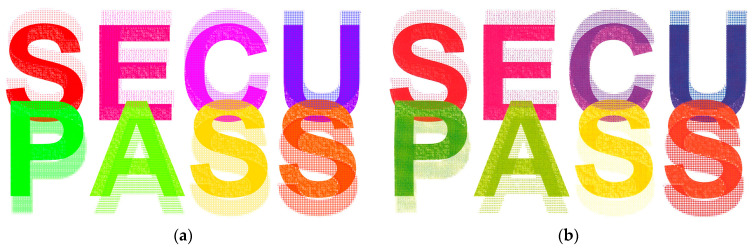
(**a**) Shows the original security graphic and (**b**) shows the result after printing and rescanning the graphic.

The last example was created to show the possibility of counterfeit protection. The example shows the original graphic and what happens to it after printing and rescanning. The graphic would be very difficult to reconstruct using vector graphics software because some shapes are created using random numbers, i.e., they are built and distributed randomly. The shadow of the first red letter S, which passes over the letter P, has lost the structure of the shape. The letters ‘P’ and ‘A’ show too much light detail from the shadow. The yellow letter ‘S’ has practically lost its shadow. The colors are completely different, especially visible in the letters ‘C’, ‘U’, ‘P’, and ‘A’. This experiment was performed with the Canon ImagePress C165. The use of different shapes, algorithms known only to the authors, and the correct distribution of shapes according to the detected hue make our proposed method a good way to protect graphics from forgery. Figure 15a shows the original security graphic and Figure 15b shows the result after printing and rescanning the graphic.

**Figure 16:**
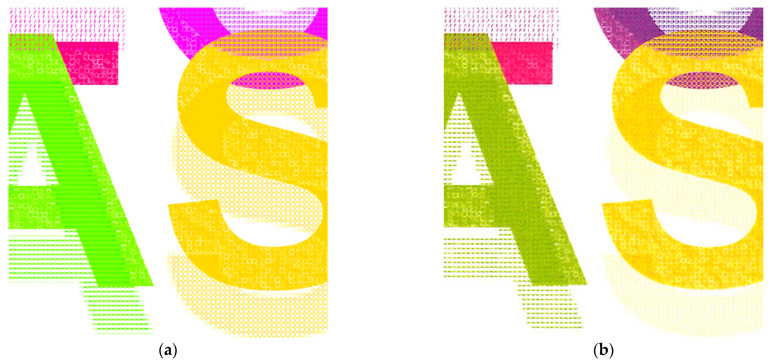
Enlarged details of Figure 15; (**a**) original security graphics and (**b**) enlarged details of rescanned graphics.

Figure 16a,b show the enlarged details of security graphics from those displayed in Figure 15.

## 4. Discussion and Conclusions

This work shows that by converting from CMYK to RGB and then to the HSB system, it is possible to manipulate pixels more easily and influence their replacement by different vector shapes depending on the hue. We managed to represent 20 different vector shapes by replacing 20 hue intervals and one additional interval for black. When converting from the CMYK system to the HSB system, deviations and anomalies were encountered. These anomalies are particularly pronounced for greens and blues, as shown in the paper. These hues are an area where research could be expanded. Several other conclusions follow from this research. It is possible to manage different hues and replace the pixels representing their values with different vector shapes. There are numerous graphic elements that can be used as a replacement for square pixels. Thus, it is possible to fill the image with different elements that change the shape of the pixels. This conclusion could be used for protection against counterfeiting, including in art and in the economic sense.

Pixel manipulation creates great value in creating security graphics for printed documents. Pixel size can also be controlled so that vector shapes can be reduced so that they are not visible to the human eye, but only with the aid of a magnifying device. When scanning and reprinting, the structures of the various vector shapes that replace the pixels would be completely lost. The appearance of the pixels displayed in this way is simply impossible to reproduce without the knowledge of all the algorithms used in the work. In addition to the individualized shapes to replace standard pixel shapes, algorithms to convert to and from different color systems also contribute, and there are few programs that allow the use of three-color systems in one document. Furthermore, even if the algorithm is known, additional measurements, analysis, and corrections are required at certain intervals to achieve the desired result.

There are several software solutions for manipulating an image or its parts. At the time of writing, we have not yet found one that can process so many hues simultaneously and in different ways. The original image can be rendered quite differently, either on an artistic basis or on a strictly programmable basis. Moreover, a multicolor graphic can be represented in innumerable ways by changing the parameters or the vector shape that represents a particular hue.

## Figures and Tables

**Chart 4:**
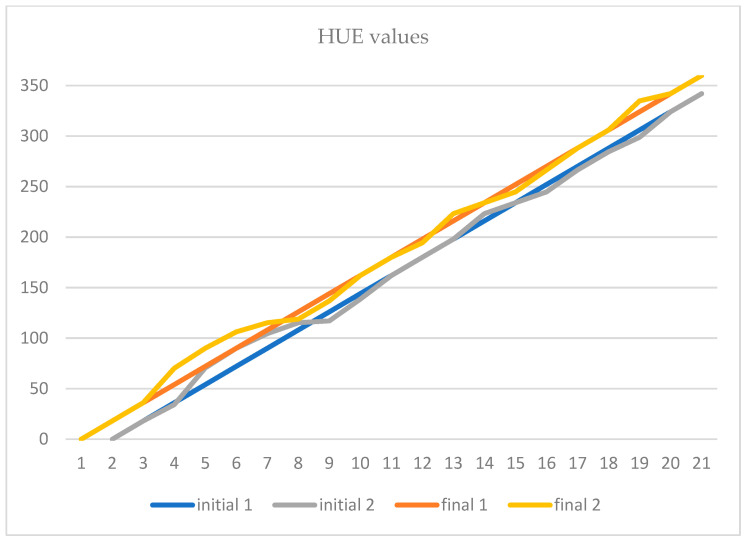
Shows the difference between the initial and final values of the hue interval determined mathematically by the program, and those measured in the case of a regular shape distribution on the color wheel.

**Table 1:** Shows the differences in the correlations of C, M, Y on hue (H) with respect to the mathematically determined angle relative to the measured values for five different color settings.

Mathematecally				Euroscale Coated V2				Uncoated Fogra 29			
H	C	M	Y	H	C	M	Y	H	C	M	Y
0	0	100	100	0	0	94	87	0	0	98	99
18	0	70	100	18	0	80	88	18	0	78	100
36	0	40	100	36	0	46	89	36	0	41	100
54	0	10	100	54	2	4	89	54	3	1	91
72	20	0	100	72	30	0	100	72	25	0	100
90	50	0	100	90	53	0	100	90	45	0	100
108	80	0	100	108	65	0	100	108	58	0	100
126	100	0	90	126	68	0	100	126	60	0	100
144	100	0	60	144	66	0	91	144	58	0	90
162	100	0	30	162	62	0	49	162	57	0	46
180	100	0	0	180	58	0	14	180	52	0	12
198	100	30	0	198	71	12	0	198	69	13	0
216	100	60	0	216	86	55	0	216	92	61	0
234	100	90	0	234	92	70	0	234	100	75	0
252	80	100	0	252	90	73	0	252	98	77	0
270	50	100	0	270	78	77	0	270	86	80	0
288	20	100	0	288	53	78	0	288	67	81	0
306	0	100	10	306	27	80	0	306	42	81	0
324	0	100	40	324	0	92	0	324	3	94	0
342	0	100	70	342	0	95	52	342	0	100	56
**Japan Web Coated**				**Photoshop 5**				**Web Coated V2 U.S.**			
H	C	M	Y	H	C	M	Y	H	C	M	Y
0	0	98	89	0	0	87	99	0	0	99	100
18	0	88	90	18	0	78	98	18	0	84	100
36	0	52	93	36	0	51	95	36	0	47	100
54	2	7	93	54	1	11	93	54	2	4	99
72	31	0	100	72	26	0	93	72	25	0	100
90	53	0	100	90	46	0	90	90	48	0	100
108	65	0	100	108	55	0	88	108	60	0	100
126	67	0	100	126	56	0	86	126	62	0	100
144	65	0	90	144	53	0	68	144	60	0	95
162	63	0	53	162	46	0	41	162	57	0	51
180	61	0	15	180	38	0	16	180	52	0	13
198	74	14	0	198	70	15	0	198	65	15	0
216	86	67	0	216	86	58	0	216	81	61	0
234	90	82	0	234	100	78	0	234	87	76	0
252	88	84	0	252	100	81	0	252	84	78	0
270	78	87	0	270	84	77	0	270	69	79	0
288	56	89	0	288	58	70	0	288	45	82	0
306	30	91	0	306	37	68	0	306	21	84	0
324	0	96	0	324	6	87	0	324	0	96	0
342	0	98	55	342	0	90	62	342	0	99	61

**Table 2:** The values from Chart 1 obtained mathematically, with intervals of 18 degrees and the values for the correct representation of shapes by each segment of hue.

	H Mathematically (Degrees)	H Mathematically (Values 0–1)	Interval (Degrees)	H Displaying Correctly (Degrees)	H Displaying Correctly (Values 0–1)	Interval (Degrees)
1	0–18	0–0.05	18	0–18	0–0.05	18
2	18–36	0.05–0.1	18	18–36	0.05–0.01	36
3	36–54	0.1–0.15	18	34.2–70.2	0.095–0.195	36
4	54–72	0.15–0.2	18	70.56–90	0.196–0.25	19.44
5	72–90	0.2–0.25	18	90–106.2	0.25–0.295	16.2
6	90–108	0.25–0.3	18	104.4–115.2	0.29–0.32	10.8
7	108–126	0.3–0.35	18	115.2–118.8	0.32–0.33	3.6
8	126–144	0.35–0.4	18	117–136.8	0.325–0.38	19.8
9	144–162	0.4–0.45	18	138.6–162	0.385–0.45	23.4
10	162–180	0.45–0.5	18	162–180	0.45–0.5	18
11	180–198	0.5–0.55	18	180–194.4	0.5–0.54	14.4
12	198–216	0.55–0.6	18	198–223.2	0.55–0.62	25.2
13	216–234	0.6–0.65	18	223.2–234	0.62–0.65	10.8
14	234–252	0.65–0.7	18	234–244.8	0.65–0.68	10.8
15	252–270	0.7–0.75	18	244.8–266.4	0.68–0.74	21.6
16	270–288	0.75–0.8	18	266.4–288	0.74–0.8	21.6
17	288–306	0.8–0.85	18	284.4–306	0.79–0.85	21.6
18	306–324	0.85–0.9	18	298.8–334.8	0.83–0.93	36
19	324–342	0.9–0.95	18	324–342	0.9–0.95	18
20	342–360	0.95–1	18	342–360	0.95–1	18

## Data Availability

Not applicable.

## Supplementary Materials

The following supporting information can be downloaded at: https://www.mdpi.com/article/10.3390/jimaging9060106/s1, Figure S1: The deviations of the shape distribution depending on detected hue for Euroscale coated V2 color setting; Figure S2: The deviations of the shape distribution depending on detected hue for Japan web coated color setting; Figure S3: The deviations of the shape distribution depending on detected hue for Photoshop 5 color setting; Figure S4: The deviations of the shape distribution depending on detected hue for U.S. web coated V2 color setting; Code S1: Postscript code for Figure 6; Code S2: Postscript code for Figure 8.
