# Supplementary material for: Manipulating Pixels in Computer Graphics by Converting Raster Elements to Vector Shapes as a Function of Hue
Source: J Imaging. 2023 May 23;9(6):106. doi: 10.3390/jimaging9060106 (PMC10299400; doi:10.3390/jimaging9060106)
Supplement: Supplementary file 1 [file jimaging-09-00106-s001.zip › jimaging-2355961-supplementary/Supplementary Figures.pdf]

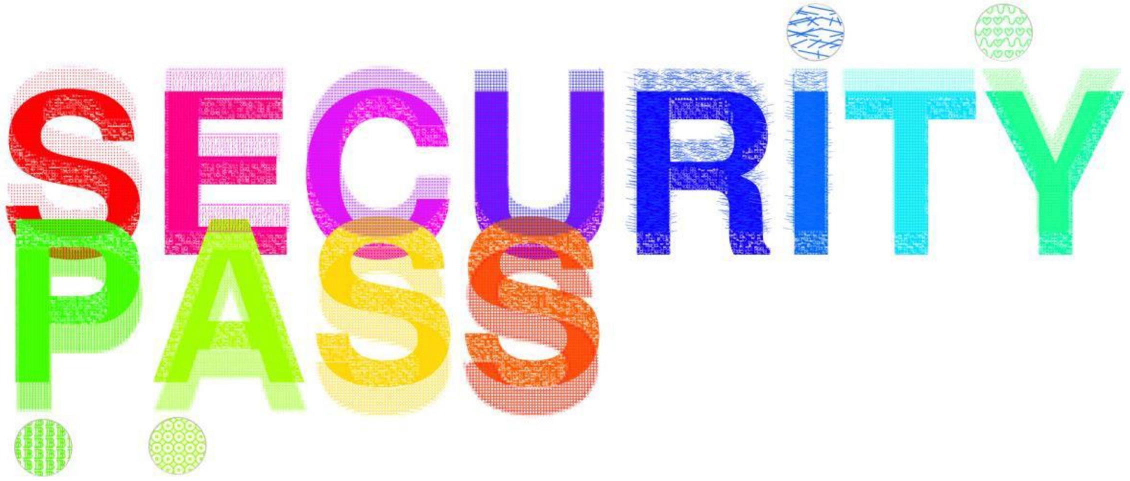

Figure S1. The deviations of the shape distribution depending on detected hue for Euroscale coated V2 color setting.

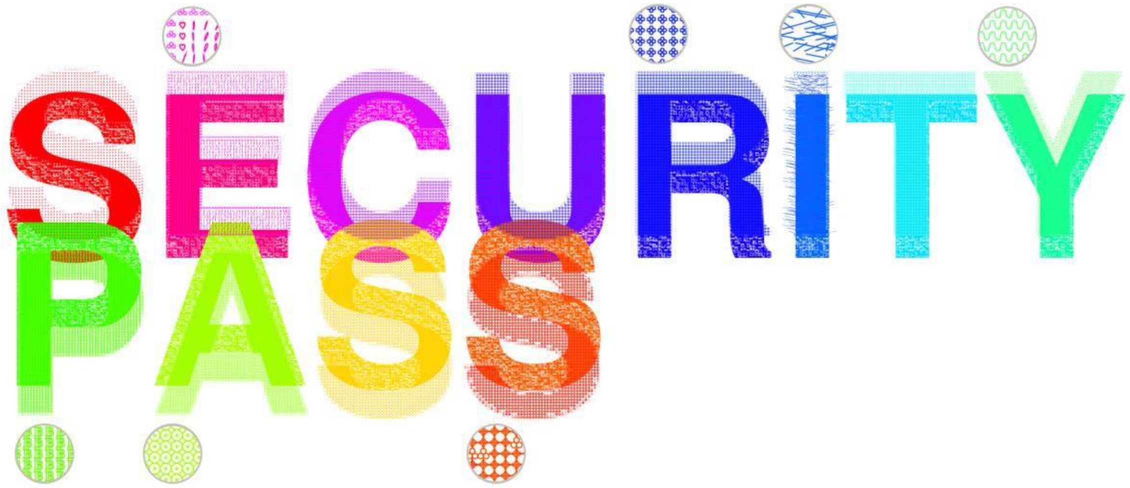

Figure S2. The deviations of the shape distribution depending on detected hue for Japan web coated color setting.

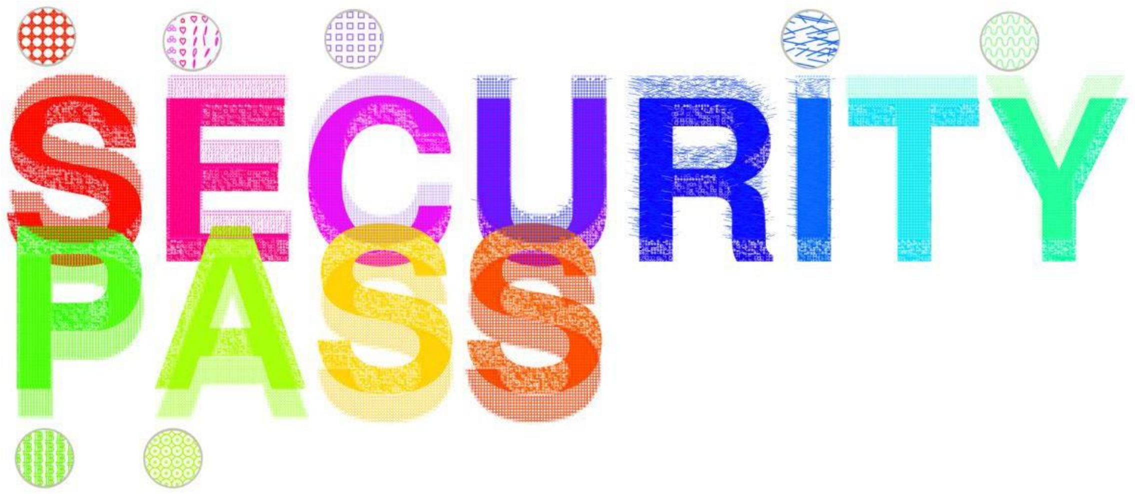

Figure S3. The deviations of the shape distribution depending on detected hue for Photoshop 5 color setting.

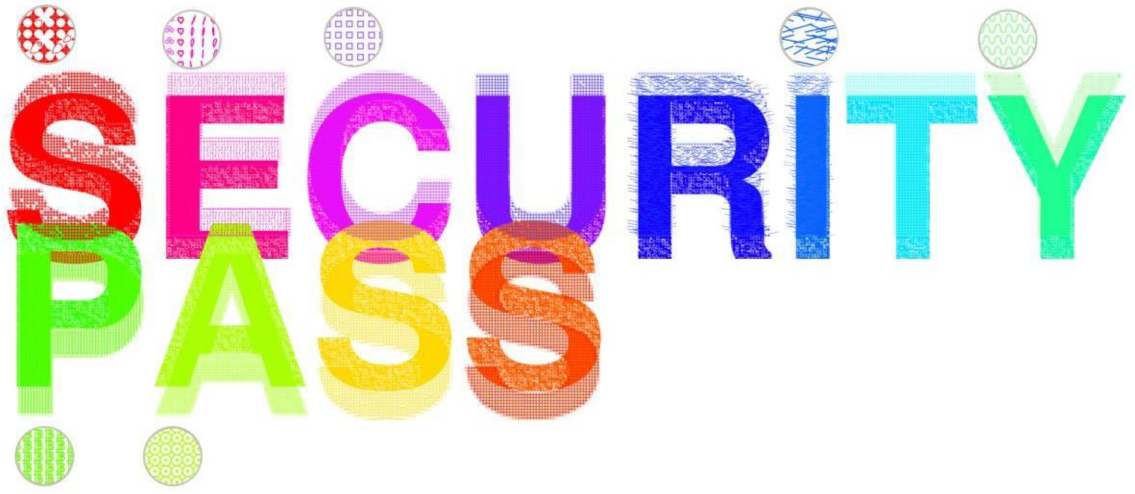

Figure S4. The deviations of the shape distribution depending on detected hue for U.S. web coated V2 color setting.
